# Teamwork skills, shared mental models, and performance in simulated trauma teams: an independent group design

**DOI:** 10.1186/1757-7241-18-47

**Published:** 2010-08-31

**Authors:** Heidi Kristina Westli, Bjørn Helge Johnsen, Jarle Eid, Ingvil Rasten, Guttorm Brattebø

**Affiliations:** 1 Department of Psychosocial Science, University of Bergen, Bergen, Norway; 2 Department of Anaesthesia and Intensive Care, Haukeland University Hospital Bergen, Bergen, Norway

## Abstract

**Background:**

Non-technical skills are seen as an important contributor to reducing adverse events and improving medical management in healthcare teams. Previous research on the effectiveness of teams has suggested that shared mental models facilitate coordination and team performance. The purpose of the study was to investigate whether demonstrated teamwork skills and behaviour indicating shared mental models would be associated with observed improved medical management in trauma team simulations.

**Methods:**

Revised versions of the 'Anesthetists' Non-Technical Skills Behavioural marker system' and 'Anti-Air Teamwork Observation Measure' were field tested in moment-to-moment observation of 27 trauma team simulations in Norwegian hospitals. Independent subject matter experts rated medical management in the teams. An independent group design was used to explore differences in teamwork skills between higher-performing and lower-performing teams.

**Results:**

Specific teamwork skills and behavioural markers were associated with indicators of good team performance. Higher and lower-performing teams differed in information exchange, supporting behaviour and communication, with higher performing teams showing more effective information exchange and communication, and less supporting behaviours. Behavioural markers of shared mental models predicted effective medical management better than teamwork skills.

**Conclusions:**

The present study replicates and extends previous research by providing new empirical evidence of the significance of specific teamwork skills and a shared mental model for the effective medical management of trauma teams. In addition, the study underlines the generic nature of teamwork skills by demonstrating their transferability from different clinical simulations like the anaesthesia environment to trauma care, as well as the potential usefulness of behavioural frequency analysis in future research on non-technical skills.

## Background

Members of trauma teams are expected to share a common goal, and to synchronise individual skills in interdependent collaboration in order to provide safe and efficient patient care [1]. Although team members are sufficiently trained individually, teamwork skills have traditionally been less emphasised in medical training [2]. The knowledge that fatal errors due to 'human factors' can occur in 70-80% of medical mishaps has led to growing interest in medical teams' cognitive and interpersonal skills, such as leadership and communication, which are referred to as 'non-technical skills' [3]. Such ability has shown to have a critical role in maintaining safety, especially for individuals working in teams in high-risk domains, and would thus be essential for trauma teams [4]. In Norway, 'Better & Systematic Trauma Care Foundation' (BEST) has introduced a systematic approach to improving medical management in trauma teams nationwide [5].

A promising approach to identifying medical teamwork skills has been developed by researchers at the University of Aberdeen [6]. The Non-Technical Skills behavioural marker system was developed from incident analyses, team observation, and attitude surveys of effective teamwork skills, first for anaesthetist, and later for other clinicians [7]. The system measures five areas of teamwork: coordination; information exchange; use of authority and assertiveness; assessing capabilities and; supporting behaviour. This system has shown good reliability and validity when field tested on different clinical situations and in both low and high fidelity simulations [8,9].

In addition to the medical domain, research on teamwork effectiveness has long been used by the military, where team leadership and effective coordination can literally be a matter of life or death. From a series of studies conducted on military tactical teams, it has been reported that effective team performance under a high workload is dependent on the team members' ability to apply a shared understanding of the task, the structure of the team, and the team members' roles within it. This proposed beneficial cognitive construct is referred to as a shared mental model (SMM) [10], and are assumed to enable team members to predict task needs and the actions of other team members by offering an immediate and internalised understanding of how team members coordinate behaviour and choose different actions without explicit demands being made for coordination. Although the concept has been studied in military teams, so far very few have applied this concept to a medical domain. This is surprising, given that the observation of behavioural markers of shared mental models has been particularly advocated in contexts in which user-system interaction is highly structured, where error detection is of particular interest and in domains where verbalisation is a normal part of task performance which is the case for trauma teams [11,12]. The concept of shared mental models may therefore be particularly applicable in trauma settings, complementing the construct of teamwork skills.

Thus, the first objective of this study was to field test and to validate the teamwork skills system by observing and assessing trauma team simulations. The present study offers a new methodological approach to team analysis compared with other studies [13]. An accumulative (moment-to-moment) quantitative approach was applied in this study, in contrast to a single global assessment of team performance. Based on theory and prior research the following hypothesis will be tested: the more favourable medical management outcomes will be associated with a higher frequency of good teamwork skills, while the opposite will happen in teams with higher frequencies of poor teamwork skills displayed.

The second objective was to assess whether shared mental models would help to explain differences in team performance. From previous research on teamwork in healthcare, we assumed that, in order to maximise performance, the interdependent nature of performance in the emergency medical domain would require a high level of implicit coordination and mutual understanding among team members [14]. Thus, a second hypothesis was tested: teams with a high frequency of behavioural markers indicating shared mental models will display superior medical management outcomes, over and beyond what is found for teamwork skills.

## Methods

A total of 27 Norwegian trauma teams from hospitals participating in the BEST-programme participated in the study. Each team consisted of five or six medical professionals, amounting to a total of 139 clinicians. Each trauma team included a surgeon (team leader), an anaesthesiologist, an anaesthetic nurse, an emergency medical nurse and a radiographer. The video recordings included in the study were originally recorded for training purposes. They were selected from more than 100 audio-video recordings based on: a) audio quality and b) video quality. The trauma training simulations were organised and carried out in local hospitals. The hospital's team set-up, procedures and equipment were used and team members acted out their own professional roles in the scenarios, thus increasing the ecological validity of the study. The same simulation scenarios were used for all teams and were based on real patient cases. The teams were expected to have the expert knowledge and skills to execute established ABCDE-procedures. The video recordings varied in length and the number of observed behavioural categories was therefore registered as an average per minute. The teamwork behaviour of each individual team member was rated, before the observed teamwork behaviours for each team were summed up [15]. The teams were observed using Noldus Observer XT - a software system that enables observable behaviour to be scored and subjected to quantitative analysis [16].

The measurement of the teams' medical management was based on two outcomes. First, two experienced clinicians independently scored the video recordings to estimate a Performance Score based on an *a priori *set of medical criteria: Airways, Breathing, Circulation and haemorrhage control, Disability, and Environment and exposure, known from ATLS [17]. In this, the teams should ascertain the patient's status, and bring her to either CT or surgery. The clinicians were selected on their medical expertise and personal experience from trauma teams, thus they were well familiar with the procedures and the simulated patient cases. They received rater training to provide a common frame of reference for evaluating each of the targeted performance criteria incorporated in the study. Each of the five criterion outcomes was rated separately on a five-point Likert scale (range: 1 = very poor to 5 = very good) before being summarised to form a composite performance index (range: 5 = very poor to 25 = very good). In addition, the two subject matter experts also rated a global measure of the teams' technical management for each simulating trauma team, called Medical Management. The overall medical management measured only the technical skills exhorted by the trauma teams and was rated from 1 = very poor to 5 = very good.

Teamwork skills were measured using a revised version of the ANTS system, as shown in Table 1.

**Table 1:** Teamwork skills from the ANTS system

Teamwork skills	Definitions	Examples of markers of good behaviour	Examples of markers of poor behaviour
Coordination	Managing synchronous and/or simultaneous activities to align the pace and sequencing of others' contributions with goal accomplishment	Confirms roles and responsibilities of team members	Does not involve team in task

Information exchange	Giving and receiving the knowledge and data necessary for team coordination and task completion	Gives situation updates/reports key events	Fails to express concerns in a clear and precise manner

Use of authority	Observable behaviour of leading the team and/or the task (as required) or accepting a non-leading role when appropriate	Gives clear orders to team members	Does not allow others to put forward their case

Assessing capabilities	Providing physical, cognitive and emotional help to team members and seeking help from others when necessary	Notices that a team member does not perform task to expected standard	Does not pay attention to the performance of other members of the team

Supporting behaviours	Providing physical, cognitive and emotional help to team mates, and seeking help from others when necessary	Anticipates when colleagues will need equipment or information	Asks for information at difficult/high workload time for someone else

Behaviours indicating shared mental models were measured with the Anti-Air Teamwork Observation Measure [ATOM; 20]. Indicators were a) Provide information (e.g. provides information *before *being asked), b) Provide support (e.g. provides assistance *before *being asked), c) Team initiative (e.g. provides guidance or makes suggestions to team members), and d) Communicating situational awareness (e.g. provides situation updates). Three psychologists trained in observing and rating the frequency of teamwork skills and shared mental model indicators independently scored the video recordings in random order. Contrary to global ratings which have been criticized for not obtaining valid results caused by for example rater errors [18], or observation biases, frequency ratings have been considered to be more reliable [19], and could be performed by raters with human factors knowledge, as was the case in this study.

The ANTS and ATOM behavioural rating systems were revised and adjusted to fit the context and tasks of a trauma team, based on theoretical work on ANTS, initial observations of approximately 20 trauma teams in training simulations, and, 4 semi structured interviews with experienced anaesthetists and intensive care workers working in Norwegian hospitals. The data from observations and interviews were only used to modify already existing behavioural indicators from the original systems. Firstly, behaviours that were not suitable for the environment in which trauma teams operate were excluded from the revised measure. Behavioural markers from the original measure like *"Observes that a team member has returned from sick leave and enquires about their general health"*, and *"Joins established team without ascertaining their capabilities" *were excluded since the observed teams were of a temporary kind, and performed in a simulated scenario. Secondly, the scoring formats of ANTS and ATOM were revised to index the moment-to-moment behaviour of the individual team members [20]. Thirdly, the skills categories of the ANTS system had behavioural markers indicating both good skills (e.g. *Provide assistance when requested*) and poor skills (e.g. *Uses a dismissive tone in response to requests from others*). The poor behavioural markers were in our study grouped into the following sub-categories: Poor Coordination, Poor Use of authority, and Poor Supporting Behaviour. From ATOM, the poor behavioural markers that indicate a lack of shared mental models in the team were grouped in one skills category: Poor Communicating situational awareness. Finally, each teamwork skill was given defining examples of behaviours, based on the original measures to ensure the inter-rate reliability of the observers.

An independent group design was used to explore whether teams with higher levels of teamwork skills and behavioural markers of shared mental models would receive higher performance scores than teams with lower levels of such behaviour [21]. This was tested using *t*-tests for independent samples. A bi-variate correlation analysis with Pearson's correlation coefficient was performed to assess the associations between teamwork skills, behavioural markers of shared mental models and team performance. In the subsequent analysis, teamwork skills and behavioural markers of shared mental models that correlated significantly with performance scores were entered in a multiple regression analysis to predict team performance outcomes. A multiple regression approach was chosen in order to assess if behavioural markers of shared mental models would augment the effect of teamwork skills (i.e. hypothesis 2). The variables were included in the equation if they fulfilled the inclusion criteria (p < .05). The results from the regression analyses were based on Adjusted R Square due to the relatively small sample size [22].

The Norwegian Social Science Data Service approved the application for consent to use the video recordings used in the study. Each team was only classified at hospital level. Measurements were not calculated for individual team members, since it was the teams' concerted performance that was studied. It was not necessary to apply to Regional Committee for Medical and Health Research Ethics as we did not collect any data concerning health issues in this study.

## Results

The inter-rater reliability for the two independent observers for the performance score index was .72, and .74 (both *p's *< .05) for the Medical Management measure, based on intra-class correlation. A correlation analysis between the performance score and the medical management measure showed a correlation of the two performance measures of .90 based on Pearson's correlation (*p *< .01). In order to create two equally large comparison groups a median split of the two measures was performed, resulting in 14 higher-performing teams and 13 lower-performing teams when measured by Performance Score, and 18 higher-performing teams and nine lower-performing teams based on their Medical Management. The inter-rater reliability for the three independent observers of the teamwork skills and behaviours indicating shared mental models was .72 (*p *< .05) based on intra-class correlation.

To explore the first hypothesis, bi-variate correlations were examined. The results in Table 2 reveal a positive correlation between the teamwork skill information exchange r (26) = .34, *p *< .05 (one-tailed) and team performance, whereas the teamwork skill poor coordination correlated negatively r (26) = -.36, *p *< .05 (one-tailed) with team performance. Finally, the teamwork skill of supporting behaviour correlated negatively with team performance r (26) = -.37, *p *< .05. Correlations between the different teamwork skills varied from small to moderate (Table 2).

**Table 2:** Correlations between teamwork skills and performance outcomes in trauma teams (N = 27).

Teamwork skills	Performance Score	Medical Management	**1**. Coordination	2. Poor Coordination	**3. Info**. exchange	4. Use of authority	5. Poor use of authority	6. Assessing capabilities	7. Supporting behaviour
1. Coordination	-.06	.25	-						
2. Poor Coordination	-.23	-.36***	-.11	-					
3. Information exchange	.11	.34***	.60**	-.42*	-				
4. Use of authority	.03	.26	.42*	-.31	.52**	-			
5. Poor use of authority	-.15	.05	.22	.02	.03	.26	-		
6. Assessing capabilities	.11	-.04	-.13	-.03	-.14	-.02	-.04	-	
7. Supporting behaviour	-.37***	-.14	.50**	.09	.23	.31	.66**	-.17	-

8. Poor supporting behaviour	.17	.19	.17	-.03	.30	.35***	.55**	-.06	.43*

*p *< .05 ** *p *< .01 *** *p *(one-tailed) < .05

To explore the second hypothesis, correlations were examined between behavioural markers of shared mental models and team performance. The behavioural marker from ATOM; provide information correlated positively, r (26) = .51, *p *< .01, with performance outcomes, whereas the behavioural marker from ATOM poor communicating situational awareness correlated negatively, r (26) = -.40, *p *< .05, with team performance outcomes. The two behavioural markers of communicating situational awareness and provide support correlated strongly, while the associations among the other behavioural markers of shared mental models were rather small (Table 3).

**Table 3:** Correlations between shared mental model indicators and performance outcomes in trauma teams (N = 27).

Shared mental model indicators	Performance Score	Medical Management	1. Provide information	2. Communicating SA	3. Poor communicating SA	4. Provide support
1. Provide information	.09	.51**	-			
2. Communicating situational awareness	.14	.20	.07	-		
3. Poor communicating situational awareness	-.40*	.04	.41*	-.42*	-	
4. Provide support	-.13	.04	.06	.81**	-.04	-
5. Team initiative	-.06	.12	.07	.50**	-.09	.50**

*p *< .05 ** *p *< .01

To further examine differences between higher and lower performing teams, t- tests were performed with both performance indicators (Performance Score and Medical Management score). The analysis revealed that higher-performing teams showed a significantly lower frequency of the teamwork skill of supporting behaviour [t (26) = -2.01; *p *< .05], and exchanged significantly more information [t (26) = 1.80; *p *< .05] compared with lower-performing teams, as shown in Table 4. The magnitude of differences in means (mean difference = -.29, 95% CI: -.59 to .01) was large (eta squared = .14) for supporting behaviour, and (mean difference = .67, 95% CI: -.10 to 1.43) was also large (eta squared = .14) for information exchange. Higher-performing teams also provided significantly more information [t (26) = 2.99; *p *< .01] and communicated situational awareness significantly more [t (26) = -2.19; *p *< .05], than lower performing teams, thus demonstrated more behaviours that indicated shared mental models compared with lower-performing teams. The magnitude of differences in means (mean difference = 1.14, 95% CI: .36 to 1.93) was very large (eta squared = .26) for providing information, and (mean difference = .21, 95% CI: -.22 to .63) was large (eta squared = .16) for communicating situational awareness.

**Table 4:** Means, standard deviations and significant values for teamwork skills and SMM-indicators in higher and lower-performing teams (N = 27).

	Medical Management Skills				Performance Score			
	Higher team performance (N = 18)		Lower team performance (N = 9)		Higher team performance (N = 14)		Lower team performance (N = 13)	
	$\bar{x}$	SD	$\bar{x}$	SD	$\bar{x}$	SD	$\bar{x}$	SD
*ANTS-Teamwork skills*								
Coordination	1.14	0.38	0.92	0.50	1.05	0.48	1.10	0.38
Poor coordination	0.01	0.02	0.04	0.08	0.01	0.02	0.03	0.07
Information exchange	2.78	0.93	2.11	0.87*	2.66	1.03	2.44	0.88
Use of authority	1.64	0.51	1.41	0.59	1.80	0.60	1.77	0.59
Poor use of authority	0.07	0.11	0.06	0.08	0.05	0.08	0.08	0.12
Assessing capabilities	1.80	0.55	1.50	0.56	0.04	0.08	0.25	0.52
Supporting behaviour	0.60	0.35	0.72	0.49	0.50	0.38	0.79	0.37*
Poor supporting behaviour	0.05	0.08	0.02	0.03	0.05	0.09	0.03	0.03
*SMM - Indicators*								
Provide information	2.83	0.93	1.68	0.96**	2.54	0.92	2.34	1.25
Communicating situational awareness	1.51	0.47	1.23	0.57	1.50	0.52	1.37	0.50
Poor communicating SA	0.50	0.33	0.46	0.46	0.35	0.28	0.63	0.40*
Provide support	2.30	0.66	2.16	0.93	1.95	0.67	2.11	0.64
Team initiative	1.88	0.55	1.62	0.83	0.35	0.20	0.37	0.26

* *p *< .05; ** *p *< .01

In order to examine the hypothesised superiority of behaviours indicating shared mental models in relation to teamwork skills, a series of multiple regression analyses were performed with performance outcome variables. Based on the correlation analysis, the teamwork skills information exchange and poor coordination were entered into the equation in Step 1, with performance score as an outcome variable. The results from the first equation produced no significant model, In Step 2, controlling for the teamwork skills information exchange and poor coordination, the unique contribution of the shared mental model behaviour of offering information was determined. The second equation produced a significant model that explained 23% of the variance in team performance and made a statistically significant contribution to the prediction of team performance β = .51 (F = 8.93; *p *< .01).

In the multiple regression analysis with medical management as an outcome variable, the teamwork skill supporting behaviour was entered into the equation in Step 1, while poor communicating situational awareness was entered in Step 2, controlling for supporting behaviour. The first equation produced no significant model, while the second produced a significant model, where a behavioural marker of a lack of shared mental models in the team (poor communicating situational awareness) explained 13% of the variance in team performance, β = -.40 (F = 4,80; *p *< .05).

## Discussion

The findings of this study lend empirical support to the significance of teamwork skills, indicating that specific teamwork skills and behavioural markers of shared mental models are associated with team performance, thus partly meeting the aims of this study. The results indicate that by improving the teamwork skills of different groups of clinicians it is possible to improve the medical management of such teams. The present study also demonstrates an overlap between teamwork skills needed in anaesthesia and trauma care. Specific teamwork skills and behavioural markers were successfully transferred from one environment to another, indicating the generic nature of these behavioural categories. Furthermore, the present study underlines the potential significance of using frequency ratings rather than global ratings of teamwork behaviour. High frequencies of poor teamwork behaviour were negatively associated with performance outcomes, while high levels of good teamwork skills were positively related to performance. A unique feature of this study is that specific indicators of shared mental models were significantly related to performance in trauma teams, over and above specific teamwork skills. These findings support Cannon-Bowers and colleagues' emphasis on shared mental models as an implicit coordinating mechanism in high-performing teams [5].

The study lends partial support to the first hypothesis in that, some of the specific teamwork skills proposed by the ANTS model, poor coordination and information exchange were associated with trauma team performance. Although poor coordination was not able to explain the differences between higher and lower-performing teams, a significant difference emerged in relation to information exchange, where higher-performing teams showed more information exchange than lower-performing teams. This result supports findings that information exchange is important for effective teamwork and task allocation [23,24]. In trauma teams, information exchange is particularly important because of the interdependent nature of the team processes. The distinct roles and responsibilities require specific and timely information in order to prevent foreseeable adverse events [25].

Contrary to expectations, a negative association was found between the teamwork skill of supporting behaviour and team performance. Supporting behaviour was also observed less frequently in the higher-performing teams than in the lower-performing teams. One possible explanation may be that supporting behaviour occurs as a result of a workload capacity problem in teams and should therefore not be associated with effective team performance alone. A request for help may not reflect objective task needs as much as an unwarranted dependency, which could have counterproductive effects in critical situations. It has been suggested that by only focusing on the frequency of help requests without a corresponding examination of capacity and workload, it will not be possible to discriminate between legitimate and illegitimate needs for help [26]. Hence, this issue should be studied in more detail in order to determine what kinds of supporting behaviour are positively associated with high-quality team performance.

According to our second hypothesis, indicators of shared mental models (Offering information and Poor communicating situational awareness) explained 23% and 13% of the variance in performance outcomes, respectively. This is interesting, given that most studies of teamwork in healthcare have not paid attention to the shared mental model construct. It is worth noting that, although some of the teamwork skills proposed was associated with performance outcomes, they did not explain the variance in performance outcomes. This could indicate, as theoretical research has suggested, that shared mental models are needed to utilise team members' teamwork skills and that information exchange is a particularly crucial mechanism in excellent teams [23,27,28]. This is in line with Undre and colleagues, who reported that medical teams were more prone to error due to poor communication in teams with low levels of shared mental models of the team's roles [14]. It has been suggested that communication and language problems are a root cause of accidents in both aviation and healthcare [24,29], and differences in communication style between nurses and physicians are seen as a contributory factor to communication errors [30]. The results of our study indicate that communication problems may be explained by a lack of shared understanding among team members about their respective roles, tasks and objectives. Enhancing team members' understanding of the other members' roles across different medical specialties (i.e. cross-role training) could be potentially efficient to improve cooperation, if appropriately applied [14,31].

Some possible limitations of this study are worth mentioning. First of all, the simulation training may have represented an artificial situation that could have affected the teams' behaviour. However, there is reason to believe that this was not the case since the teams were selected from various Norwegian hospitals, the training situations were based on real trauma cases and the teams were complete, performing their normal tasks in their own trauma rooms with their own equipment and protocols. Secondly, there is a possibility that the process of revising the two measures used to assess teamwork may have been a threat to the construct validity. Based on the high correlations between some of the team work skills, there is a possibility that some of the team work skills were not enough nuanced, and therefore did not explain performance differences between the observed trauma teams. The high correlations could also imply that some of the teamwork skills are in fact not separate constructs, but complimentary facets of the construct teamwork. Future research should explore this issue in more detail. There is also a possibility that the behavioural markers used in the study do not reflect all important teamwork skills in this type of team. However, research confirms an overlap between teamwork skills in intensive care units, surgical teams and other high-risk domains such as aviation, leading us to argue that the most relevant skills are also applicable to trauma teams [8]. There is also a possibility that when revising the original systems in order to be used in moment-to-moment observation, we could have lost important nuances of teamwork skills, which could alter the results. We would recommend that some o the behavioural markers of teamwork skills and SMMs should bed more nuanced in future studies, to avoid a strong overlap between some of the behavioural markers, as was the case in this study Finally, this study does not address the causal relationship between performance and teamwork skills or shared mental models. An alternative explanation to the findings of this study could be that high performing teams have more capacity to also be good team players, rather than that the better team players easier obtain a good medical result. However, we assume, based on a extensive research on team and performance in other domains (e.g. aviation) that teamwork skills are important indicators of a teams overall performance. Secondly, the results of the regression analyses in this study show quite clearly that particular teamwork skills have the ability to explain a large degree of variance in medical management.

In conclusion, the present study provides new empirical evidence of the significance of teamwork skills and shared mental models in healthcare that replicates and builds upon previous research. To our knowledge, this is the first empirical assessment of the relationship between teamwork skills, indicators of shared mental models and performance in simulating a trauma team scenario. Our results suggest that the effectiveness of trauma teams could be significantly increased by their developing communication and information exchange skills. Although distinct teamwork skills and indicators of shared mental models explained differences in the medical management of the observed teams more research is needed to determine critically important teamwork skills that should be assessed and developed in trauma teams. In addition, the construct of shared mental models should be explored further, as it is reasonable, based on the results of this study, to suggest that it could be applied in and be useful for trauma teams.

## Competing interests

The authors declare that they have no competing interests.

## Authors' contributions

All authors have read and approved the final manuscript. Design of the study was performed by HKW, IR, BHJ, JE and GB. Data collection and synthesis was completed by HKW and IR. Manuscript preparation was performed by HKW, JE and BHJ. Final proofing of the manuscript was by HKW, JE, BHJ and GB.
